# Evolution of Wearable Devices in Health Coaching: Challenges and Opportunities

**DOI:** 10.3389/fdgth.2020.545646

**Published:** 2020-12-02

**Authors:** Mohammed Tahri Sqalli, Dena Al-Thani

**Affiliations:** Information and Computing Technology Division, College of Science and Engineering, Hamad Bin Khalifa University, Qatar Foundation, Doha, Qatar

**Keywords:** human-computer interaction, human-AI interaction, wearable technologies, health coaching, digital medicine, health informatics

## Abstract

Wearable devices hold an enormous potential in contributing to an improved global health. The availability, non-invasiveness, and affordability of those systems make them promising candidates to transform the standard of care for health coaching. These wearable devices are now considered as versatile coaching systems. Patients who wish to improve their health and well-being refer to wearables for tracking and quantifying their improvement. The timeliness of the “wearable device as a health coaching enabler” field of research will inevitably know a prominent growth in the upcoming years. This growth is expected to stem from both the computing and the medical fields. In this perspective article, we list the potential challenges as well as the opportunities of this newly born field from an interdisciplinary perspective. We mainly focus on both the computing and healthcare perspectives. We also chart guidelines for the healthcare research community that is willing to get involved in the computing field to harness the benefits of wearable devices.

## 1. Introduction

The use of wearable technologies in providing health coaching for patients is becoming an imminent application of computing in healthcare. We define a wearable device to be any computing device equipped with the necessary sensors to measure, process, or analyze one or many health indicators for the user wearing it (1). Wearable devices or “wearables” range from wristband and smartwatches to chestbands and other textile-based sensors. Health coaching, however, is defined as an ongoing feedback loop between an intelligent system and a user. The feedback is mostly effective to the user when it is based on the context and the environment. The aim of health coaching is to support the user achieve a certain health goal (2). The potential benefits of harnessing these technologies in the health sector also bring an important share of challenges and questions to be answered. These challenges lie in the responsibility of guaranteeing the precision, reliability, and availability of those health coaching systems for the patients using them. Moreover, the majority of those systems suffer from poor accuracy of their internal machine learning models (3, 4). These models are essential as they establish the link between the sensors of the device and the health indicators of the patient. These questions raised and others that surge push the computing research community to adopt different strategies to address these new challenges. International health institutions are responding to those challenges by establishing regulating bodies; two of the United Nations (UN) bodies, mainly the World Health Organization (WHO) and the International Communication Unit (ITU), have reacted to this forthcoming change in healthcare by founding a focus group dedicated to Artificial Intelligence for Health (AI4H) in July, 2018 (5). The United States Food and Drug Administration (FDA) is also mandating several additional criteria for these wearable devices to qualify as being approved health coaching systems (6). Among the strategies to enable a more accurate health coaching for patients, one is the referral to algorithm training using health practitioners' expertise (2). Another strategy consists of using neural networks and other similar classifiers in order to develop wearable devices' enabled health coaching systems (7).

In this perspective article, we list and illustrate in details the unique research opportunities along with the future challenges of wearable technologies as enablers to health coaching. This perspective article also aims to propose guidelines for the computing research community working in the healthcare field. These proposed guidelines help develop effective wearable devices dedicated for health coaching. The guidelines and perspectives proposed in the discussion section are concluded from the body of research of several existing biomedical wearable devices used for primary healthcare research (8). These devices are also approved and granted clearance by the United States Food and Drug Administration (FDA). In order to support the guidelines and perspectives proposed, we provide illustrative examples along with each guideline.

## 2. Wearable Devices as Enabling Tools for Health Coaching: Between Challenges and Opportunities

The use of wearable devices as enabling tools for health coaching poses a myriad of questions. These questions are timely pertinent as the line between computing and healthcare is becoming more and more blurred (9). The questions that arise concern the effectiveness of these wearable devices in comparison to their clinical counterparts (6). The effectiveness of the health coaching programs that these devices curate are being under scrutiny when compared to a health coaching program supervised by a healthcare practitioner. Moreover, physical limitations and the inherent wearable device's margin of error in terms of measurements limit the use of these devices in clinical settings (2). This is due to the small form factor dictated by those devices. In health coaching, accurate measurements are vital for the success of the patient's coaching program. Thus, an inaccurate measurement by the device might cause considerable disturbances to the health coaching program's normal flow. Finally, with all these concerns being at the forefront of the design process, new paradigms (2, 10–12), design considerations (6, 13, 14), and methodologies (15, 16) stimulate, inspire, and advance the field of computing for healthcare.

### 2.1. Challenges for Wearable Devices as Enabling Tools for Health Coaching

1. Accuracy and precision of the raw data measured through the device's sensors (4): Wearable devices contribute preponderously toward the continuous monitoring of patients' health indicators. This monitoring is done by the device's sensors. However, this contribution is only beneficial when the devices are overmonitored and recalibrated regularly by health experts. A wearable device that bases its health coaching feedback on uncalibrated erroneous data will certainly mislead the user. In some cases, like for patients with a chronic disease, the consequences may be detrimental, if not lethal to the patient (4, 6).
2. Compatibility with the clinical ecosystem (17): This refers to the difference in the standards of measuring, processing, and storing the wearable's data. This difference usually exists between the clinical private databases of patients and the wearable device. Compatibility issues of consumer grade wearable devices dedicated for health coaching arise when their design does not account for the basic elements of clinical compliance. Accounting for clinical compliance requires following rigorous FDA regulations and standards (3, 6). The process of clinical compliance focuses on guaranteeing unified channels of signal acquisition and transfer across the clinical ecosystem. Examples of wearable health coaching devices that overcame this specific challenge are wearable electrocardiograms (ECGs). Wearable ECGs have a unified standard for sensors manufacturing (Electrodes), as well as a unified standard of measuring, communicating, and transferring the measured ECG across the clinical and non-clinical ecosystems (4, 8).
3. Shortcoming of machine learning models in this domain (6): Machine learning, in the context of wearable technologies, refers to the use of the collected health indicators' data about the user. These health indicators are used as features in order to design accurate predictive and recommender models to understand the user's health-related trends. Wearable devices dedicated for health coaching follow a computational paradigm where a machine learning model drives the health coaching program. This model continuously analyzes and deduces trends from simple collected health indicators (4, 8). Health indicators vary between core body temperature, heart rate, and blood pressure and others. However, these indicators are not specialized and contextualized enough to provide an accurate personalized health coaching programs, especially for a patient with a chronic condition (2, 7).

### 2.2. Opportunities for Wearable Devices as Enabling Tools for Health Coaching

1. Convenient form factor for a high frequency of measurements (4): By a convenient form factor, we refer to both the ergonomics of the device (e.g., its ease of wearability), as well as its usability (e.g., its ease of use and the possibility of frequent usage). To satisfy the ergonomics aspect, manufacturers opt for a compact form factor. The compact form factor for wearable devices dedicated for health coaching allows both the flexibility and personalization needed for patients to maintain adherence to their health coaching programs (2). Monitoring patients health indicators through wearable devices opens the possibility for a high frequency of measurements with long time intervals. This allows for a detailed identification of patients health trends. Moreover, the compact form factor of wearable health coaching devices ensures the flexibility of monitoring, guiding, and coaching patients, while they remain in their ambulatory environments, including their residences and their workplaces. This advantages hospitals and health coaches to not be overwhelmed by a surplus of unnecessary patient visits (6). Furthermore, this aspect diminishes healthcare costs while capitalizing on the patients' convenience by advocating for remote and tele-helthcare (2).
2. Personalized care and personalized coaching (2): The concept of personalized care refers to the possibility of adapting the healthcare program of the users to their individual needs. The ability for wearable devices to timely monitor patients health indicators allows for a uniquely personalized care and coaching to every patient using these wearable devices. Personalized care and coaching includes individualized dietary recommendations (18), as well as individualized pharmaceutical dosage (19, 20) adapted daily to the in situ health situation of the patient.
3. Efforts to integrate wearable devices in the medical ecosystem (21–23): Active research is being conducted to lay the ground and develop efficient and effective semi-autonomous wearable devices for health coaching patients. Laying the ground for this research requires the satisfaction of both the computational processing needs and the medical research needs. These requirements mainly focus on the full compatibility of the data that the wearable technologies generate with the electronic health records where the data are stored and processed.

## 3. Discussion

Wearable devices dedicated for health coaching promise an improved healthcare system. The advent of this new modality of interaction with patients pushes the research community to design innovative and effective human-wearable interaction modes. Currently, the intersection between computing and healthcare brought unique, specific, and unprecedented requirements. Therefore, the main motivation of this discussion section is to lay the fundamental guidelines to researchers and investigators in both the computational and healthcare fields to achieve a successful conception and development of wearable technologies. As the field of health coaching using wearable technologies is niche, developing computational health solutions for it demands special considerations.

Following is a compiled list of recommended guidelines for a successful human-wearable interaction design. The proposed list follows a chronological order with reference to the design process. It starts from the conceptualization of the wearable device, and ends with its manufacturing. It is noteworthy that these guidelines follow the process of three distinct ordered compliance themes. These three compliance themes are as follows: Validation compliance (this theme includes guidelines from 1 to 4), measurements compliance (this theme includes guidelines from 5 to 8), and wearability compliance (this theme includes the last three guidelines from 9 to 11). The list is curated from the body of research conducted in the field.

1. Identify one main important benefit that the wearable device for health coaching brings (6, 24): The objective behind developing a wearable device system for health coaching needs to be based on grounded research with an aim to provide benefits to the healthcare sector. The research approach needs to account for the safety of the device. It also needs to take into consideration methodologies for assessing the wearable device's effects on the patient. The device needs to eventually prove a quantified progress in terms of the user's health.
2. Gather a scientific body of research to advice on the effectiveness of the wearable device (4, 25): This allows for providing certainty and credibility to confirm any pre-proposed hypothesis with regard to the feasibility of the design. It also allows to have an early idea about the monitoring capabilities of the device before being developed.
3. Plan for viable methodologies to validate the wearable health coaching system (4, 6): Validating the wearable system includes drafting methodologies for the collection of the patients' data. It also includes drafting measurable metrics to track the wearable system performance. Validating methodologies also needs to guarantee that the ground truth of the wearable device is calibrated appropriately for each user.
4. Experimenting with different study designs and clinical trials to validate and develop the wearable device (4): It includes brainstorming as well as using the trial and error methodology to draft a study design/clinical trial as early as in the conceptual design process. This allows for opening the space to discuss the safety of the users involved in the study, the efficacy of the wearable health coaching device, as well as the diversification of subjects involved in the study. Moreover, presenting the study design to an institutional review board provides rich feedback from both the medical and the computing experts.
5. Account for the clinical compliance of the wearable health coaching device during the design process (5, 26, 27): Accounting for the clinical compliance refers to the process of following the FDA's methodologies (or a similar institution's methodology) for validation. Among the core components of the validation criteria are measurement precision, the performance of the device in different clinical and non-clinical settings, and its compatibility with the clinical workflows and ecosystems.
6. Account for including transducer sensors: This helps in enhancing the reliability of the wearable device's measurements of the patient's biomedical metrics (6). Transducer sensors refer to sensors that are able to convert variations of physical quantities, like brightness or pressure into an electrical signal for digital data processing. These sensors are essential in the precision of taking ECGs for example.
7. Bridge the gap between the raw data collected by the wearable device sensors and the wearable device output interface (28): This is done by developing analytical methodologies that process and analyze the collected health indicators' data. These analytical methodologies then use machine learning models and established medical ground truths to cross-validate the user's data with the medical recommendations. The analytical models most often need to work in the background to hide the complexities of the system from the user.
8. Periodically evaluate the wearable device's system performance (5, 26, 27): Evaluation of the wearable device's performance is done using pre-set metrics during the design process. Evaluation is also done with reference to the health experts' recommended ground truths, cross-validation methods, as well as the recommended confidence intervals for each periodic evaluation. These evaluation methodologies eventually contribute toward the credibility and quality assurance of the wearable device.
9. Guaranteeing the possibility for both the continuous and episodic monitoring for the patient's health indicators (15): This ensures that the wearable health coaching device is compliant to different segments of patients, as well as to different health coaching needs. While some chronic diseases require a continuous monitoring for the patient's health indicators, other chronic diseases require periodic monitoring. To illustrate this, heart rhythm irregularities require a continuous monitoring of the heart rhythm due to the fact that these irregularities happen infrequently and abruptly (4, 6). However, for diabetes monitoring there is no need for a continuous monitoring as the glucose concentration in the blood needs hours to vary significantly.
10. Develop active and adaptive notification mechanisms for when the wearable device's system fails (4, 29): System failures stem from different sources. It is therefore valuable to inform the user about the source or the reason of the failure. This allows the user to understand from where to proceed in order to address that system failure. Conventional ECG systems apply this guideline in order to help users take correct ECGs. For example, the “lead-off” notification in medical ECG machines notifies the medical technician who is taking the ECG to check if all the 12 leads are placed properly on the patient's body. Moreover, other types of notifications are incorporated after ECG recording like the beats and voltage intervals calculation notifications. These calculation notifications are the results of the medical experts advising the system on the gold standards of a healthy person's heart rate with respect to the heart rate of the patient.
11. Ensure the privacy of the user and the security of the wearable health coaching system (30): Wearable devices dedicated for health coaching patients face both the challenge of ensuring the privacy of the patient, as well as the challenge of the security of the data that is constantly generated by the device. Moreover, wearable device face an inherent cybersecurity weakness as they need to constantly transfer the data they generate to a master controlling device (either a smartphone or a service provider server) (31). Instructions and guidelines on how to safeguard the privacy of the patient and the security of the wearable health coaching device are highlighted in the requirements of the Health Insurance Portability and Accountability Act (32).

## 4. Conclusion

### 4.1. Concluding Remarks

Throughout this perspective article, we have discussed the challenges and hurdles facing research in computing for healthcare. We have focused the discussion on the challenges facing the use of wearable devices for health coaching specifically. These challenges are unique to this emerging research area. Wearable devices for health coaching bring numerous novel research questions, and yet also bring answers to some previously raised concerns. Among these challenges are the challenge of privacy, precision of sensors measurements, and the challenge of compatibility with medical ecosystems.

Along with these challenges, considerable responsibilities await the research community. These responsibilities are unique to the field of computing in healthcare. Health coaching is an extremely sensitive field that impacts directly the lives of patients, whereby the cost of an error might be detrimental to the patient's life. This article attempted to prove that for each major challenge, several novel opportunities direct this new research field. Thus, we have discussed the forthcoming opportunities in healthcare with the advent and affordability wearable devices for health coaching. As these are becoming more and more an integral aspect of patients' daily life, the medical and computing research communities are finding innovative ways in order to harness this source of data. So far, these research efforts culminated in the design of wearable devices that have a form factor compelling for a convenient high frequency measurements. Moreover, wearable devices are leading the way toward a personalized care and personalized health coaching. The essence of personalized care is focused around internal machine learning models that curate a personalized health coaching programs for each patient using the device.

In the discussion section, this perspective article summarized, in the form of research best practice recommendations, the research process needed to validate the development of a wearable device for health coaching. These research best practice traverse the whole journey starting from the design of the health coaching device until its end development.

### 4.2. Future Directions

The pressing global demand for improving the quality, availability, and affordability of healthcare will unavoidably require a continuously precise and detailed monitoring of patients using wearable devices for health coaching. Wearable technology holds immense potential in the near future for being able to flatten the cost curve of healthcare. The research field of computing in healthcare has the potential to guide the clinical evolution in how healthcare is delivered in the near future. The richness of collected data because of different compact ubiquitous computing modalities, which are affordable and available to large segments of the population, will stimulate prompt timely diagnosis. It will also unveil hidden emergency events in patients' health. Computing in healthcare will also encourage the development of novel artificial intelligence models suitable for wearable devices. These artificial intelligence models will be key initiators in the management and prediction of variances in the patient's health. Finally, this evolution in healthcare will certainly involve an increasing number of researchers in different fields to create an interdisciplinary community focusing its research efforts in developing solutions for wearable devices for health coaching.

## Author Contributions

MT and DA-T contributed the conception and brainstorming of the article. MT organized the structure of the article and wrote the manuscript. All authors contributed to manuscript revision, read, and approved the submitted version.

## Conflict of Interest

The authors declare that the research was conducted in the absence of any commercial or financial relationships that could be construed as a potential conflict of interest.
